# Enhancing Biomass and Lipid Production in *Messastrum gracile* Using Inorganic Carbon Substrates and Alternative Solvents for Lipid Extraction

**DOI:** 10.3390/life15030407

**Published:** 2025-03-05

**Authors:** Wanida Pan-utai, Soisuda Pornpukdeewattana, Wilasinee Inrung, Theera Thurakit, Penjit Srinophakun

**Affiliations:** 1 Department of Applied Microbiology, Institute of Food Research and Product Development, Kasetsart University, Bangkok 10900, Thailand; ifrtet@ku.ac.th; 2 School of Food Industry, King Mongkut’s Institute of Technology Ladkrabang, Bangkok 10520, Thailand; soisuda.po@kmitl.ac.th (S.P.); taenggkwa.1995@gmail.com (W.I.); 3 Department of Chemical Engineering, Faculty of Engineering, Kasetsart University, Bangkok 10900, Thailand; fengpjs@ku.ac.th

**Keywords:** biomass, extraction, lipid, microalgal growth, productivity

## Abstract

Microalgae show promise as a biomass and bioproduct for applications in various industries. The cultivation of microalgae plays a crucial role in optimizing biomass yield and bioproduct accumulation. The provision of inorganic carbon substrates substantially enhances microalgal growth and lipid biosynthesis, resulting in marked increases in the production of biofuels and other bioproducts. This study examined biomass and lipid accumulation in *Messastrum gracile* IFRPD 1061 under inorganic stress conditions, previously unreported. *M. gracile* IFRPD 1061 was subjected to varying conditions of inorganic carbon substrates, 1–3 g·L^−1^ sodium carbonate and bicarbonate concentration, to enhance biomass and lipid accumulation. Optimal productivity levels were observed with sodium bicarbonate addition of 3 g·L^−1^ and 1 g·L^−1^ for biomass and lipids, resulting in productivities of 392.64 and 53.57 mg·L^−1^·d^−1^, respectively. Results underlined the effectiveness of sodium carbonate and bicarbonate as inorganic carbon sources for stimulating microalgal growth and enhancing the production of high-value products. The extraction of lipids from freeze-dried biomass of *M. gracile* IFRPD 1061 demonstrated optimal yield using methanol/hexane solvents compared with the control experiments. Lipid extraction yields using methanol/hexane were 42.18% and 46.81% from oven-dried and freeze-dried biomass, respectively. Lipids extracted from oven-dried *M. gracile* IFRPD 1061 using methanol/hexane/chloroform solvents indicated the potential of methanol/hexane as a solvent for lipid extraction from dry microalgal biomass using an ultrasonic-assisted technique. This study contributes valuable insights into maximizing biofuel and bioproduct production from microalgae, highlighting *A. gracilis* as a promising candidate for industrial applications.

## 1. Introduction

Global energy demand is continually increasing. Microalgae provide a sustainable option to meet the growing energy needs while also significantly reducing the environmental impact [1]. Innovative biotechnological methods show great potential by utilizing microalgae as a renewable source of biofuel feedstock [2]. Microalgae have emerged as a highly promising option for the generation of biofuels, functional food ingredients, and nutraceuticals, attracting considerable interest [3]. Numerous microalgal species with pharmacological and biological qualities have been extensively explored for their potential as high-value products [4]. Microalgae are a clean and renewable alternative to liquid fossil fuels and offer cost-effective solutions to address environmental concerns by efficiently converting atmospheric carbon dioxide into valuable products such as carbohydrates, lipids, and bioactive metabolites [5,6]. Their transformative potential makes them a compelling choice for sustainable and eco-friendly solutions [5]. Strains of green freshwater microalgae including *Scenedesmus* sp., *Crucigenia* sp., *Spirogyra* sp., *Oscillatoria* sp., *Chlorella* sp., *Chlamydomonas* sp., *Euglena* sp., *Cladophora* sp., *Microcystis* sp., *Messastrum* sp., and *Pediastrum* sp. have shown potential for biodiesel production [7]. Microalgae grow rapidly, exhibit high photosynthetic efficiency, and utilize land effectively, resulting in significant biomass and lipid production for biofuel applications [8].

Microalgae are one of the most promising alternative sustainable feedstocks for the production of lipid-based biofuels due to their high lipid accumulation capacity, rapid growth rate, and high photosynthetic efficiency [9]. Increasing the accumulation of lipids in microalgae enhances the economic viability of biofuel production. Genetic and metabolic engineering methods, as well as cultivation regulation strategies, have been summarized in multiple recent reviews to improve microalgal cell and lipid accumulation or productivity [10]. The cultivation process is important for effective lipid production from microalgae [11,12] to maximize industrial biofuel production by optimizing the growth process and the production of valuable byproducts [9]. The optimization of nutrient and environmental conditions plays a significant role in the improvement of microalgal cell growth and lipid production. Several factors influence the growth of microalgae and their lipid production, including sources of carbon and nitrogen, temperature, light intensity, and pH levels, which vary depending on the species of microalgae [13,14,15,16]. Microalgae can be grown under different carbon sources that depend on modes of cultivation as photoautotrophic, heterotrophic, and mixotrophic [17]. Photoautotrophic cultivation offers several advantages including a higher growth rate attributed to increased photosynthetic efficiency [18]. This mode also presents a lower risk of biological contamination, making it a safer option. Photoautotrophic scalability is relatively easy, allowing for potential expansion and increased production capacity [19].

A staged cultivation system that leverages nitrogen starvation stress is one of the most dependable approaches for increasing microalgal lipid production. Implementing environmental stress at a large scale is more feasible in practical terms for industrial applications [9]. Integrating environmental stress during the nitrogen starvation phase of microalgal cultivation significantly boosted microalgal lipid production in large-scale staged cultivation processes [20]. Effectively optimizing and regulating the entire stress-integrated staged cultivation process is crucial for the commercialization of microalgal lipid production [21]. Nutrient depletion and nutrient stress have been extensively studied during microalgal cultivation for their capacity to induce lipid production [22]. Other factors such as nitrogen depletion, phosphate starvation, salinity stress, and metals (e.g., iron, magnesium) as stress-inducers have all been identified as stimulants [23]. A previous report indicated that a high concentration of sodium bicarbonate at 160 mM inhibited cell growth but stimulated lipid production in *Chlorella vulgaris* [24]. Sodium bicarbonate served as the source of inorganic carbon for carbon dioxide capture via *Chlorella vulgaris* [25].

Lipid extraction is an essential stage in the downstream processing of biodiesel production from microalgae. However, methods of commercial lipid extraction from microalgae require careful consideration and attention [26]. One of the most popular methods is solvent extraction using combinations of nonpolar and polar solvents [27]. Another commonly used technique for extracting lipids from microalgae is the Bligh and Dyer method, which involves a combination of chloroform (nonpolar), methanol (polar), and water [28]. This method has been successfully used for lipid extraction from various biological samples [29]; however, the demand for safer and biocompatible solvents is increasing due to concerns about biosafety issues associated with extraction solvents [30]. Chloroform is classified as a hazardous solvent, and effective alternatives are required for lipid extraction. The moisture content of microalgae is effectively reduced through the drying process, which plays a vital role in maintaining the stability and preservation of bioactive components. This process is crucial for the development of pharmaceutical formulations, medicinal agents, and nutraceuticals [31]. Therefore, drying microalgal biomass is also commonly applied to prepare it for large-scale production as a key factor in lipid extraction yield.

This research assessed the impact of sodium carbonate and sodium bicarbonate as inorganic substrates on biomass production and lipid accumulation of *Messastrum* sp. Lipid extraction methods were optimized by evaluating various biomass preparations and solvent combinations to determine the most beneficial conditions.

## 2. Materials and Methods

### 2.1. Microalgae Identification

The microalga *Messastrum gracile* (formerly *Ankistrodesmus gracilis*) (Chlorophyta) IFRPD 1061 was obtained from the Institute of Food Research and Product Development, Kasetsart University, Thailand. *Messastrum gracile* IFRPD 1061 was prepared in a modified BG 11 medium for biomass productivity following Pan-utai, et al. [32] composed of nutrients in g·L^−1^ as 0.75 NaNO_3_, 0.02 K_2_HPO_4_, 0.038 MgSO_4_·7H_2_O, 0.054 CaCl_2_·H_2_O, 0.009 C_6_H_8_O_7_, 0.003 (NH_4_)_5_ Fe(C_6_H_4_O_7_)_2_, 0.001 EDTA.Na_2_, 0.03 Na_2_CO_3_ and micronutrients in mg·L^−1^as 1.340 H_3_BO_3_, 0.905 MnCl_2_·4H_2_O, 0.110 ZnSO_4_·7H_2_O, 0.195 Na_2_MoO_4_·2H_2_O, 0.040 CuSO_4_·5H_2_O, and 0.025 Co(NO_3_)_2_·6H_2_O. All chemical media were purchased from Merck (Darmstadt, Germany), KemAus (N.S.W., Australia), and Ajax Finechem Pty Ltd. (Auckland, New Zealand). *M. gracile* IFRPD 1061 was cultured in a clear glass photobioreactor in chamber equipment, with a temperature of 30 °C, light and dark cycles of 16:8 h, and fluorescent light intensity of photon flux density 162 µmol m^−2^s^−1^. Carbon dioxide was mixed with air at 2%, feeding continuously at a flow rate of 0.67 vvm. Cells were grown into the log phase at 7 days of cultivation, and then centrifuged at 10,000× *g* for 10 min (Model 6000, High-Speed Refrigerated Centrifuge, Kubota, Osaka, Japan) at 4 °C and then washed twice with sterilized water.

The microalgal species were identified through analysis of the 18S rRNA gene. DNA extraction from the cell collection was conducted following the instructions provided in the Genomic DNA Mini Kit (plant) (Nucleic Acid Extraction Kit, Geneaid, New Taipei City, Taiwan). The polymerase chain reaction (PCR) amplification utilized the 18S primers 63F (ACGCTTGTCTCAAAGATTA) and 1818R (ACGGAAACCTTGTTACGA) [33]. PCR was conducted in a final volume of 50 μL, with concentrations: 3 mM MgCl_2_, 0.1 mM dNTP, 0.2 µM of each primer, 1 × Taq buffer, and 2.5 U Taq polymerase (Vivantis, Shah Alam, Malaysia), along with 50 ng of template DNA. The PCR process consisted of an initial denaturation at 94 °C for 10 min, followed by 30 cycles of denaturation at 94 °C for 60 s, annealing at 55 °C for 30 s, extension at 72 °C for 45 s, and a final extension at 72 °C for 7 min using the PTC Tempo thermocycler (Bio-Rad, Hercules, CA, USA). The nucleotide sequencing was performed by commercial sequencing services (Macrogen, Seoul, Republic of Korea). The similarity of the target sequence was determined using BLAST 2.16.0 on the NCBI server (http://www.ncbi.nlm.nih.gov/BLAST/, accessed on 4 September 2024). A phylogenetic tree was constructed using the maximum likelihood method and 1000 bootstrap replications, with MEGA7 used for evolutionary analysis.

### 2.2. Microalga Inoculum Preparation

*Messastrum gracile* IFRPD 1061 inoculum was prepared in a modified BG-11 medium, as explained in Section 2.1. The microalga *M. gracile* IFRPD 1061 was cultured in a clear glass photobioreactor in chamber equipment at 30 °C, light and dark cycles of 16:8 h, and fluorescent light intensity of photon flux density 162 µmol m^−2^s^−1^. Carbon dioxide was mixed with air at 2%, feeding continuously at a flow rate of 0.67 vvm. Cells were grown to the log phase at 7 days and then harvested in the old medium by centrifugation at 10,000× *g* for 10 min (Model 6000, High-Speed Refrigerated Centrifuge, Kubota, Osaka, Japan) at 25 °C and then washed twice with sterilized water. The inoculum for experiments was prepared using a cell pellet, adjusted to a 0.2 optical density at 680 nm or cell dry weight of approximately 0.1 g·L^−1^.

### 2.3. Biomass and Lipid Accumulation Using Inorganic Supplementation

The microalgal strain *Messastrum gracile* was studied in a two-stage culturing process. In the initial stage, the microalga was cultivated in a glass photobioreactor and the cells reached the exponential growth phase on the eighth day of cultivation.

Sodium carbonate and sodium bicarbonate regulate pH levels and provide inorganic carbon (CO_2_) for photosynthesis. Initial doses of 1, 2, and 3 g·L^−1^ maintained optimal pH and carbon availability. Higher doses can enhance carbon for microalgae but may also disturb pH levels and ionic balances and cause stress or nutrient issues [34,35]. Therefore, in the subsequent stage, inorganic substrates were introduced to induce lipid accumulation. Sodium carbonate (Na_2_CO_3_, Merck, Darmstadt, Germany) and sodium bicarbonate (NaHCO_3_, KemAus, Cherrybrook, NSW, Australia) as the inducing agents were added to the culture medium at concentrations of 1, 2, and 3 g·L^−1^ of the final culture medium. The cells were then incubated in the same chamber equipment under identical controlled conditions. All experiments were conducted in triplicate. Samples were taken every 2 days up to 24 days to determine the biomass, lipid, pH, nitrate, and phosphorus during cultivation in all experiments.

### 2.4. Determination of Biomass

The dry weight of biomass cells was determined using the gravimetric method. Samples (10 mL) were filtered through GF/C filter paper (Whatman, Maidstone, UK), washed twice with distilled water, and dried at 105 °C to constant weight. The equation relating cell dry weight to optical density at 680 nm was developed to measure cell dry weight concentration during cultivation. Cells were collected every 2 days throughout the cultivation experiments and their optical density was measured at 680 nm using a spectrophotometer (SP-8001, UV-Vis Spectrophotometer, Metertech, Taipei City, Taiwan). Cell growth was assessed by establishing a correlation between cell dry weight concentration (g·L^−1^) and optical density at 680 nm, following the equation $0.6559\times OD_{680}\;$(R^2^ = 0.9018) from a previous study [32].

### 2.5. Determination of Lipid Content

Cell culture samples were collected and centrifuged at 3660× *g* for 10 min (Frontier^TM^ 2000 Multi Centrifuges, Ohaus, Parsippany, NJ, USA) and then washed twice with distilled water. Lipid content was determined following the method of Bligh and Dyer [28] with some modifications. The cells were suspended in a mixture of distilled water, methanol, and chloroform at a ratio of 0.8:2.0:1.0 and then thoroughly mixed. The mixture was carefully transferred to an ultrasonic bath (DT 100 H, Bandelin, Berlin, Germany), and the system was set to operate at a power level of 320 W and a frequency of 35 kHz. The mixture underwent ultrasonic treatment for 10 min to ensure thorough mixing. The lipid phase was then separated through centrifugation at 3660× *g* for 10 min. The lipid phase was collected, and the extraction was repeated until no color was present in the cell debris. The total lipid collected was filtered to remove any debris contamination and then dried at 80 °C until constant weight.

### 2.6. Determination of pH, Nitrate, and Phosphorus

The culture medium was separated using centrifugation at 3660× *g* for 10 min (Frontier^TM^ 2000 Multi Centrifuges, Ohaus, Parsippany, NJ, USA) and the pH value and nitrate and phosphorus concentration of the supernatant were determined. A pH meter (Lab850 Model, Schott, Mainz, Germany) was used to measure the pH during cultivation, with nitrate and phosphorus concentrations determined using colorimetric methodology according to the standard methods for the examination of water and wastewater [36]. Nitrate concentration was measured from 5 mL of the culture medium mixed with 0.1 mL of 0.1 N HCl solution. The mixture was measured for absorbance at 220 and 275 nm using a spectrophotometer (SP-8001, UV-Vis Spectrophotometer, Metertech, Taipei City, Taiwan). Sodium nitrate was used as the standard for calculating the nitrate concentration. The phosphorus concentration was determined using the vanadomolybdophosphoric acid colorimetric method. First, the vanadate–molybdate reagent was prepared by mixing solution A (25 g of ammonium molybdate in 300 mL of distilled water) and solution B (1.25 g of ammonium metavanadate in 300 mL of boiled distilled water). Then, 300 mL of 37% HCl was added and adjusted to a total volume of 1000 mL with distilled water to obtain the vanadate–molybdate reagent. For the test, 0.5 mL of the sample was mixed with 1 mL of the vanadate–molybdate reagent, followed by adding 3.5 mL of distilled water. The absorbance was then measured at 450 nm using a spectrophotometer (SP-8001, UV-Vis Spectrophotometer, Metertech, Taipei City, Taiwan). The potassium dihydrogen phosphate standard was used to calibrate the absorbance and phosphorus concentration.

### 2.7. Kinetic Parameters

The growth of the microalgal biomass and the parameters for lipid production were evaluated, while the kinetic parameters were determined using methods described in previous reports, with some modifications [37,38]. The maximum specific growth rate ($\mu_{m}$) was calculated using Equation (1), where dX/dt is the rate of microalgal growth, $\mu_{m}$ is the maximum specific growth rate of the microalga, and X and $X_{m}$ are the microalgal biomass concentration and maximum biomass concentration in the culture media, respectively.

(1) $$\frac{dX}{dt}=\mu_{m}\left(1-\frac{X}{X_{m}}\right)X$$

The volumetric rate of biomass production ($Q_{X}$) and lipid production ($Q_{P}$) were calculated using Equations (2) and (3), respectively.

(2) $$Q_{X}=\;\frac{X_{m}-X_{0}}{dt}$$

(3) $$Q_{P}=\;\frac{P_{m}-P_{0}}{dt}$$

Biomass productivity ($Q_{X}$, mg·L^−1^·d^−1^) and lipid productivity ($Q_{P}$, mg·L^−1^·d^−1^) were computed by comparing the respective variations in biomass and lipid concentrations, denoted as ($X_{m}-X_{0}$) and ($P_{m}-P_{0}$) during the cultivation period.

### 2.8. Comparison of Lipid–Solvent Extraction Methods

Lipid extraction was performed on the biomass of *M. gracile* IFRPD 1061 using various combinations of solvents to identify the most effective method for lipid recovery. A comparison was made between oven- and freeze-dried microalgal biomass. Fresh microalga was obtained from a modified BG-11 medium after 14 days of growth, collected through centrifugation at 10,000× *g* for 10 min (Model 6000 High-Speed Refrigerated Centrifuge from Kubota, Osaka, Japan) at 25 °C, and then rinsed twice with sterilized water. The cells were dehydrated in a hot air oven (Model UT6760; Thermo Scientific Heraeus Heating and Drying Ovens, Thermo Fisher Scientific Inc., Thermo Scientific, Dreieich, Germany) at 55 °C for 3 to 6 h. The freeze-dried biomass preparation involved freezing at −20 °C for 18 to 24 h, followed by freeze-drying at −50 °C at <0.06 bar pressure for 6 hours using a freeze-dryer (Labconco, Kansas City, MO, USA). The biomass preparation method was modified from Pan-utai and Iamtham [39]. The oven- and freeze-dried biomass samples were ground to 0.5 mm using a centrifugal mill grinder (ZM-1, Retsch, Haan, Germany) and stored in dark polyethylene bags at room temperature.

Lipid extraction was conducted using different ultrasonic-assisted conditions: (1) methanol/chloroform/water at a ratio of 2:2:1, (2) methanol/hexane at a ratio of 1:1, (3) hexane, and (4) methanol/hexane/water at a ratio of 2:2:1. The combination of solvents for lipid extraction was modified from a previous study [40]. Biomass concentrations were compared using oven- and freeze-dried samples at 2 g·mL^−1^. The mixtures were mixed and physically extracted to maximize the lipid yield from the microalga. The ultrasonic-assisted techniques were equipped with an ultrasonic processor (Sonic, VCX 750, Newtown, CT, USA) with a 25 mm solid probe. The samples underwent rigorous sonication at 60% amplitude with a 60 s pulse followed by a 30 s pause, at a precise frequency of 20 kHz and power of 750 W, resulting in a total extraction time of 5 min. The extracted lipids were then separated from the mixtures by centrifugation at 3660× *g* for 10 min, with this process repeated five times to ensure thorough removal of cell debris. All the experiments were carried out in triplicate. The extraction yield (%) of each extraction cycle was calculated using the weight of dried lipid (g DW) and the weight of dried biomass (g DW) using Equation (4):

(4) $$Yield=\;\left(\frac{Lipid}{Biomass}\right)\times 100$$

### 2.9. Statistical Analysis

Mean values of the parameters were calculated from triplicate experiments, and the standard deviations were determined. Statistical analysis was performed using one-way analysis of variance (ANOVA) with SPSS version 25.0 (IBM Corp., Armonk, NY, USA). Differences between treatment means were assessed using Duncan’s new multiple range test (DMRT). Statistical significance was observed at *p* < 0.05.

## 3. Results

**Figure 1:**
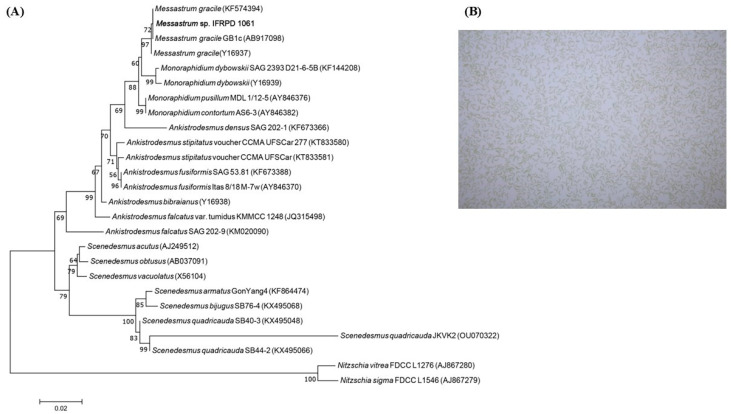
(**A**) Phylogenetic tree of 18S rRNA gene sequences of *Messastrum gracile* IFRPD 1061 inferred using the maximum likelihood method. (**B**) Microscopic observation of *Messastrum gracile* IFRPD 1061 under the light microscope (40×).

The phylogenetic tree, derived from the 18S rRNA gene sequences of *Messastrum gracile* IFRPD 1061, is presented alongside morphological observations obtained using a light microscope (Figure 1). The microalga was identified as *Messastrum gracile* IFRPD 1061 through PCR amplification and 18S rRNA gene sequencing.

### 3.1. Biomass and Lipid Accumulation of Ankistrodesmus gracilis Microalga

**Figure 2:**
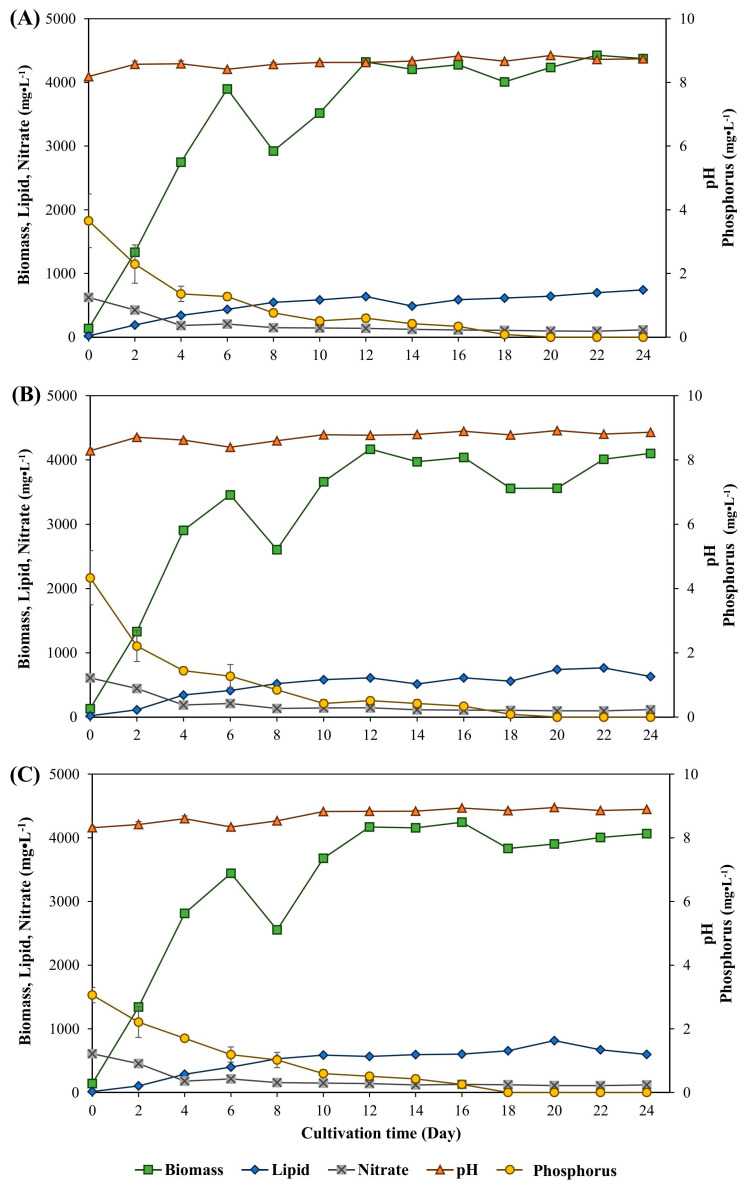
Time course of *M. gracile* IFRPD 1061 growth and lipid production under different conditions of sodium carbonate as inducing substrates at 1, 2, and 3 g·L^−1^ as (**A**), (**B**), and (**C**), respectively. All values were calculated from triplicate measurements and standard deviations.

Inorganic sodium carbonate and sodium bicarbonate substrates at different concentrations were used for *M. gracile* IFRPD 1061 microalgal cultivation. The time course of *M. gracile* IFRPD 1061 microalgal cultivation under various concentrations of sodium carbonate at 1 to 3 g·L^−1^ is shown in Figure 2. Among the various sodium carbonate substrate concentrations, the biomass concentration increased with time until day 8 of cultivation when cell growth decreased. Lipid accumulation increased with culture time. After adding 1 g·L^−1^ of sodium carbonate, the biomass increased with increasing cultivation time and reached the stationary phase after day 12 of cultivation (Figure 2A). The maximum biomass concentration was 4427 mg·L^−1^ at 22 days of cultivation, whereas the maximum lipid content was 742 mg·L^−1^ at the end of the cultivation period. For the 2 g·L^−1^ sodium carbonate addition, the maximum biomass and lipid concentrations were 4168 and 765 mg·L^−1^, respectively (Figure 2B). The maximum biomass and lipid concentration of sodium carbonate at 3 g·L^−1^ addition were 4170 and 815 mg·L^−1^, respectively (Figure 2C). The average pH values during cultivation were around 8 and did not change by more than 1. The nitrate concentration of the culture medium in all experiments decreased with increasing cultivation time and cell growth. Phosphorus concentration decreased over time in all experiments and was undetectable at the end of cultivation.

**Figure 3:**
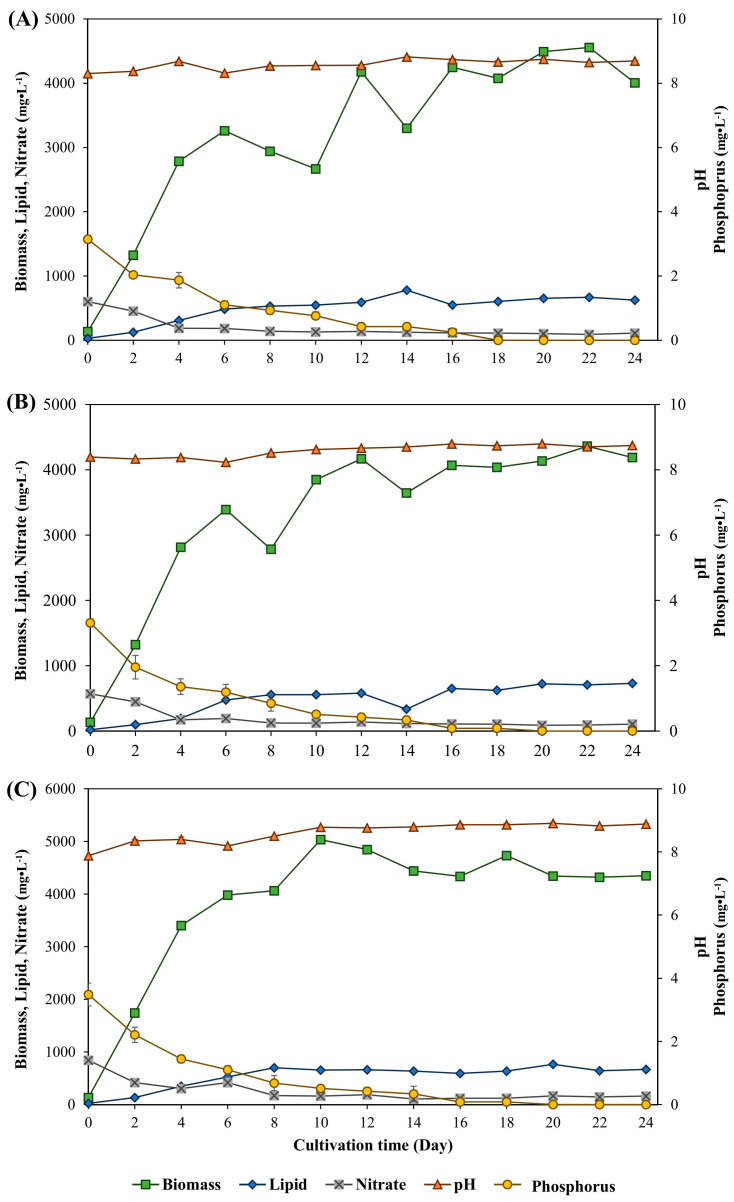
Time course of *M. gracile* IFRPD 1061 growth and lipid production under different conditions of sodium bicarbonate substrates at 1, 2, and 3 g·L^−1^ as (**A**), (**B**), and (**C**), respectively. All values were calculated from triplicate measurements and standard deviations.

Figure 3 shows the time course of *M. gracile* IFRPD 1061 growth and lipid production, with pH, nitrate, and phosphorus changing at various concentrations of sodium bicarbonate during cultivation. The microalgal biomass increased with increasing culture time and decreased during 8 to 10 days of cultivation, with added sodium bicarbonate at day 8 of cultivation. Cells increased and were stable during 16 to 24 days of cultivation as the stationary phase. Lipid contents accumulated in cell biomass during the culture period. Results showed that maximum biomass and lipid concentrations of *M. gracile* IFRPD 1061 under 1 g·L^−1^ of sodium bicarbonate supplemented were 4557 and 780 mg·L^−1^, respectively (Figure 3A). Results from an additional 2 g·L^−1^ sodium bicarbonate inorganic inducing substrate gave maximum biomass and lipid concentrations of 4362 and 730 mg·L^−1^, respectively (Figure 3B). The highest sodium bicarbonate substrate, an additional 3 g·L^−1^, gave maximum biomass and lipid contents of 503 and 700 mg·L^−1^, respectively (Figure 3C). The changes in pH were stable at approximately 8. Decreases in nitrate and phosphorus concentrations were detected during cultivation.

**Figure 4:**
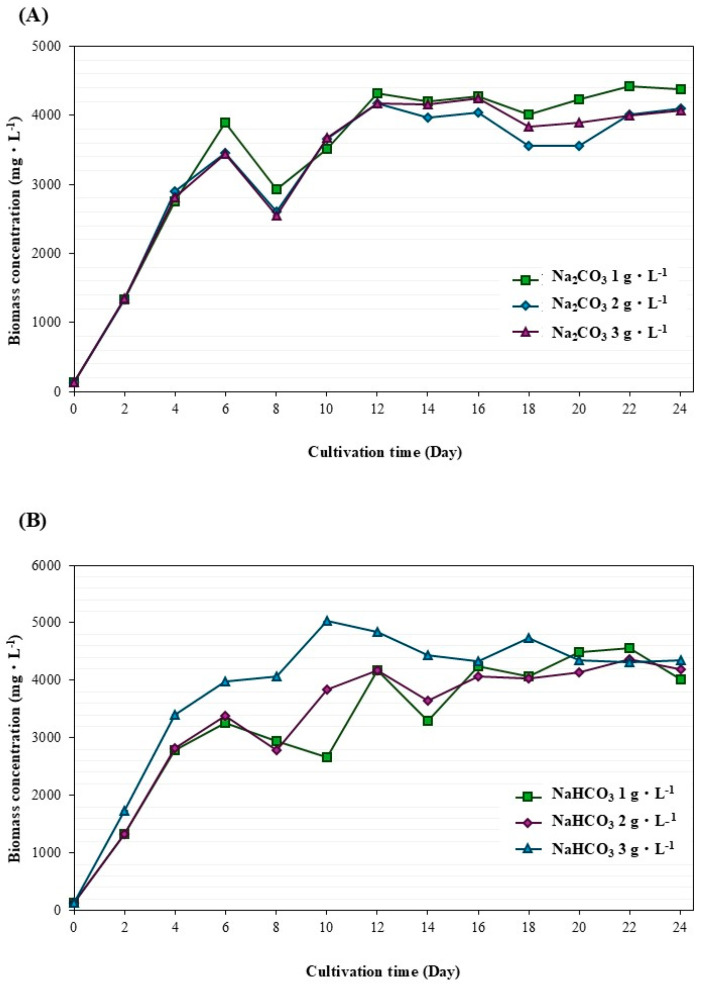
Biomass accumulation in *M. gracile* IFRPD 1061 microalgae during cultivation under various conditions, including sodium carbonate (**A**) and sodium bicarbonate (**B**). All values were calculated from triplicate measurements and standard deviations.

**Figure 5:**
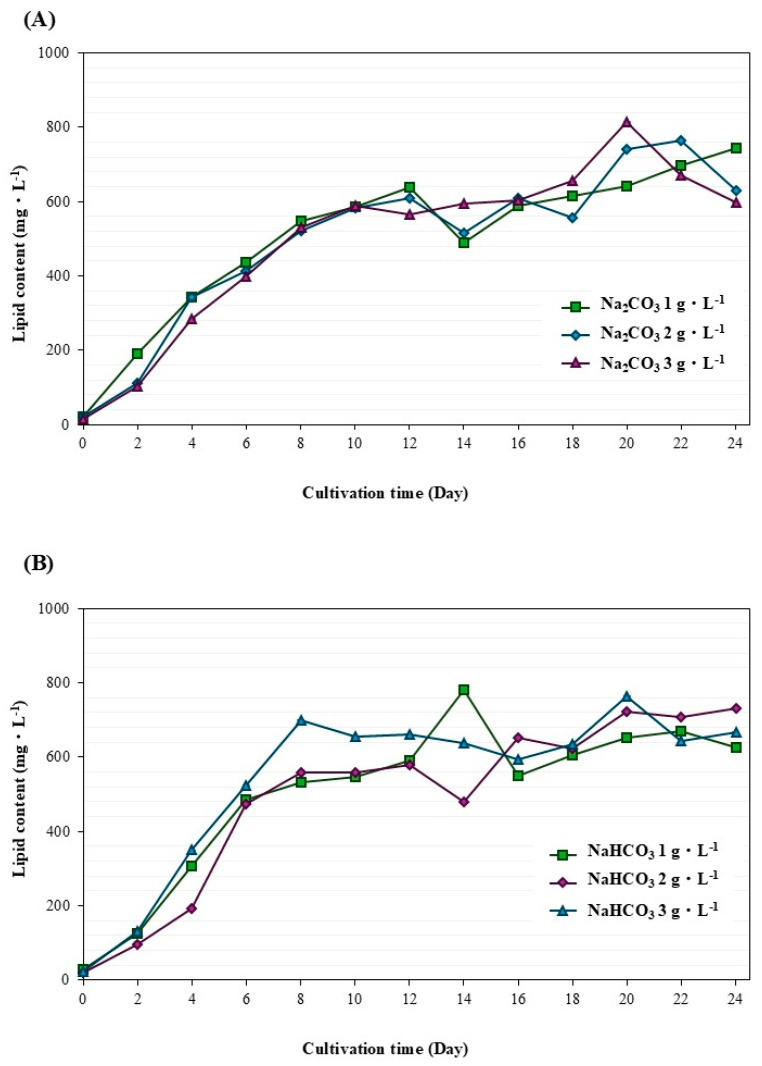
Lipid accumulation in *M. gracile* IFRPD 1061 microalgae during cultivation under sodium carbonate (**A**) and sodium bicarbonate (**B**). All values were calculated from triplicate measurements and standard deviations.

Figure 4 and Figure 5 compare the biomass concentration and lipid content under various conditions during *M. gracile* IFRPD 1061 cultivation. The highest biomass concentration was 1 g·L^−1^ of sodium carbonate, while sodium bicarbonate at 3 g·L^−1^ gave the highest biomass concentration (Figure 4). Lipid content was highest at the maximum sodium carbonate and sodium bicarbonate concentration of 3 g·L^−1^. Kinetic parameters of *M. gracile* IFRPD 1061 microalgal cultivation were achieved to maximize biomass and lipid accumulation at different conditions, with results shown in Table 1. The maximum specific growth of *M. gracile* IFRPD 1061 microalgal among various conditions ranged from 0.51 to 0.54, with insignificant differences. Biomass productivity under different conditions was highest for 3 g·L^−1^ of sodium bicarbonate supplementation at 392.64 mg·L^−1^·d^−1^. For sodium bicarbonate, the highest biomass productivity obtained at 3 g·l^−1^ was 336 mg·L^−1^·d^−1^. Lipid productivity under various conditions ranged from 30 to 54 mg·L^−1^·d^−1^. The maximum lipid productivity of 40 mg·L^−1^·d^−1^ was achieved with 3 g·L^−1^ of sodium carbonate supplementation, while the highest lipid productivity obtained from 1 g·L^−1^ of sodium bicarbonate was 54 mg·L^−1^·d^−1^ and significantly different among the other conditions. The maximum biomass accumulated during *M. gracile* cultivation was achieved from higher concentrations of carbonate supplementation, while the minimum bicarbonate concentration gave the highest lipid accumulation in the cells. Sodium carbonate and sodium bicarbonate were utilized as inorganic substrates to promote biomass and lipid accumulation in the microalga *M. gracile* IFRPD 1061. When stress was applied as an innovative technique, significant improvements were observed in cell growth and lipid accumulation in *M. gracile* IFRPD 1061.

### 3.2. Comparison of Solvents for Lipid Extraction

**Figure 6:**
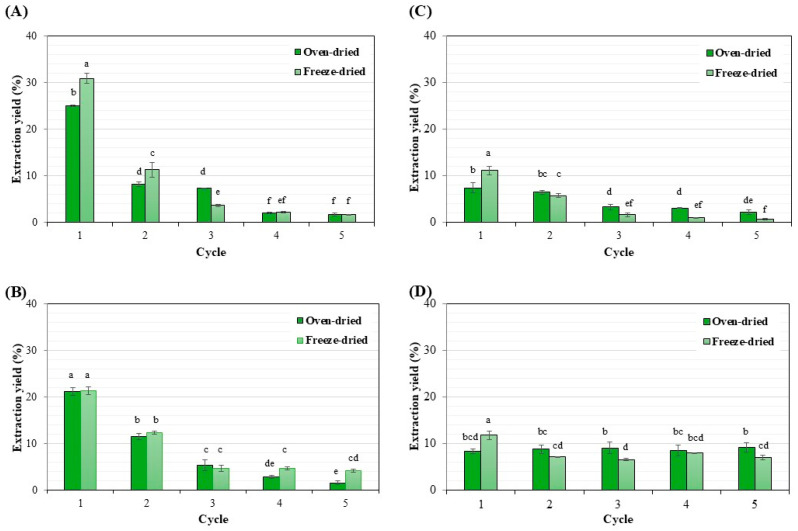
Yield of lipid extraction from *M. gracile* IFRPD 1061 under (1) methanol/chloroform/water (**A**), (2) methanol/hexane (**B**), (3) hexane (**C**), and (4) methanol/hexane/water (**D**). All values were calculated from triplicate measurements and standard deviations. Data in each group with different letters are significantly different (*p* < 0.05).

Figure 6 compares the yield of lipids extracted from the microalga under various solvents and biomass preparations across five extraction cycles. The maximum yield of lipid extracted was obtained from the first extraction cycle and decreased with repeated extraction in all experiments. Lipid extraction using mixtures of methanol/chloroform/water is a standard procedure modified by Bligh and Dyer [41] and was used as the control experiments (Figure 6A). Among oven- and freeze-dried biomass, the highest lipid yield was obtained from freeze-dried biomass during the second cycle of extraction at 31.0% and 11.3%, respectively. Using a methanol/hexane solvent mixture gave similar percentages of lipid extraction yield among oven- and freeze-dried biomass at 21% (Figure 6B). The maximum lipid yield of 11.2% from freeze-dried biomass was obtained using hexane as the sole extraction solvent (Figure 6C). The use of hexane in solvent mixtures of methanol, hexane, and water resulted in a maximum lipid yield of 12% from freeze-dried biomass during the initial extraction (Figure 6D). For repeated extractions, the yield ranged from 6.5 to 8.7%. The totals of repeated lipid extraction yields under various conditions are shown in Table 2. The highest lipid yield of 49.75% was obtained from freeze-dried biomass preparation of *M. gracile* IFRPD 1061. The preparation involved a mixture of methanol/chloroform/water using an ultrasonic-assisted method. The lipid yield from freeze-dried biomass using the mixture of methanol/hexane produced results similar to the control method, with a yield of 47%, and the difference was insignificant. Among the various extraction methods, hexane showed inappropriate results due to its low yield. The highest extraction yield from oven-dried biomass was achieved using a mixture of methanol/chloroform/water, giving results similar to the control method. Our results showed that hexane was a good alternative solvent to replace chloroform in lipid extraction from the microalga. 

## 4. Discussion

Microalgae exhibit a fast growth rate and are rich in nutrients and have high lipid accumulation capacity. They are considered promising alternative sustainable feedstocks for producing lipid-based biofuel [42]. Several significant stress conditions impact microalgal lipid production, including nitrogen, phosphate, sulfur, light, temperature, and salinity [43]. The previous report on the carbon concentrating mechanism (CCM) was studied in *Chlamydomonas* sp. as the model organism due to its known genome sequence [44]. Microalgae use an inorganic carbon (Ci) concentrating mechanism (CCM) to enhance Rubisco’s CO_2_ availability [45]. The transport of Ci through various membranes and carbonic anhydrases contributes to the accumulation of HCO^−^_3_ in the chloroplast stroma. Higher external pH increases the formation of HCO^−^_3_ ions, which are then pumped into the acidic lumen of transpyrenoidal thylakoids. There, carbonic anhydrase converts HCO_3_^−^ into CO_2_, which serves as the substrate for RuBisCO found in the pyrenoid [44,46]. Therefore, the CCM improves the photosynthetic efficiency of microalgae by raising CO_2_ concentrations. The mixotrophic mode shows high promise for microalgal growth due to its superior biomass yield, reduced biomass loss, prolonged exponential phase, and minimal photoinhibition [47]. Under mixotrophic conditions, microalgae can assimilate both inorganic and organic carbon, facilitating higher biomass yield within a shorter growth period [48]. Sodium carbonate and sodium bicarbonate are inorganic agents [49] that show potential as stress inducers to enhance biomass and lipid production during microalgal cultivation. Our results showed more effective lipid production from sodium bicarbonate than sodium carbonate, similar to results from mixotrophic *Chlorella vulgaris* microalgal cultivation [50]. The highest specific growth rate of *C. vulgaris* was 0.653 d^−1^, obtained from sodium bicarbonate 1 g·L^−1^ [49], while our results achieved a maximum specific growth rate of 0.5 d^−1^ for all concentrations of sodium carbonate and bicarbonate, concurring with a previous study [49]. Using bicarbonate supplementation to recycle reverse osmosis (RO)-rejected drinking water serves as an economical method for cultivating *C. sorokiniana* (Chlorophyta) microalgae and enhancing PUFA production [51]. Previous reports showed that the maximum biomass of *C. pyrenoidosa* was recorded at 761 mg·L^−1^ with the addition of 4 g·L^−1^ bicarbonate, which supported the synthesis of biofuel precursors. *Tetradesmus obliquus* (formerly *Scenedesmus obliquus*) and *C. sorokiniana* (Chlorophyta) achieved their highest yields of carbohydrates (339.4 mg·L^−1^) and lipids (1454 mg·L^−1^) at bicarbonate concentrations of 0.5 and 1.0 g·L^−1^, respectively [51]. The highest *Dunaliella tertiolecta* (Chlorophyta) CCAP 19/30 biomass concentration was recorded at 1.5 g·L^−1^ from a mixture of sodium bicarbonate and sodium carbonate at 5.4 g·L^−1^. Inorganic carbon in the range 0.4–13 g·L^−1^ gave high levels of lipid and protein production as primary metabolites in microalgae [52]. These results indicated that the selection of an inorganic carbon source significantly influenced metabolite production in microalgae, underscoring the critical role of carbon source variation in optimizing the cultivation process and enhancing the biochemical output of microalgal systems. A previous study showed varied morphology of *Ankistrodesmus falcatus* (Chlorophyta) in different culture media, with the cells existing individually or grouped together, and their shape was also irregular [53]. However, in *M. gracile* IFRPD 1061, the cells were single, with curved and crescent shapes that remained consistent under different conditions. Our results were similar to green algae belonging to the phylum Chlorophyta, with cells existing either as solitary entities or loosely aggregated in bundles or tufts, exhibiting needle-like, crescent shapes, or tapering narrowly at both ends, occasionally straight but generally curved [54]. The addition of inorganic supplementation during the eight days of cultivation in the log phase initially decreased biomass concentration because the cells adapted to the new nutrients and reverted to the lag phase. This disruption in nutrient availability likely affected metabolic pathways, prompting temporary growth inhibition while the cells adapted through enzyme regulation and changes in nutrient uptake efficiency [55]. However, with continued cultivation and supplementation, biomass concentration eventually increased.

Our study achieved the highest lipid productivities of 53.57 mg·L^−1^·d^−1^ using 1 g·L^−1^ sodium bicarbonate, higher than a previous study of *C. vulgaris* with 160 mM or around 13 g·L^−1^ sodium bicarbonate [24]. Sodium bicarbonate has been identified as a potential source of dissolved inorganic carbon for large-scale cultivation of *Auxenochlorella pyrenoidosa* (formerly *Chlorella pyrenoidosa*) (Chlorophyta) NCIM-2738, facilitating lutein and lipid accumulation [56]. The addition of sodium bicarbonate to microalgal cultivation provides an inorganic carbon source in the form of bicarbonate ions, which are essential for the cellular growth of microalgae [25]. Our proposed method efficiently converted CO_2_ gas and sodium carbonate solution into sodium bicarbonate using a CO_2_ bicarbonate absorber. This process facilitated the dissolution of sodium bicarbonate, which remained in the culture medium for an extended period, thereby enhancing the growth rate of microalgae [57]. The bicarbonate ions permeated the cells and were converted into CO_2_ by the enzyme carbonic anhydrase, producing the necessary CO_2_ for carbon fixation and the conversion of valuable products [58]. Sodium bicarbonate promotes fast microalgal growth and provides an efficient supply of CO_2_ to the medium, with CO_2_ and sodium bicarbonate ions utilized as the primary sources of inorganic carbon for the autotrophic growth of microalgae [59]. In mixotrophic culture conditions, sodium bicarbonate supported acetate assimilation and enhanced the putative C_4_ pathway, glyoxylate (GOX) cycle, and tricarboxylic acid (TCA) cycle in *Nannochloropsis oceanica* (Eustigmatophyceae) [60]. Therefore, inorganic carbon sources are important for microalgal autotrophic and mixotrophic cultivation modes. Numerous microalgal species have been examined for their biofuel production potential under various stress conditions. Our research on *M. gracile* revealed biomass productivity ranging from 192 to 392 mg·L^−1^·d^−1^ and lipid productivity between 29 and 53 mg·L^−1^·d^−1^. These figures significantly exceeded those observed in other species. *Phaeodactylum tricornutum* (Bacillariophyceae) produced only 0.13 mg·L^−1^·d^−1^ of biomass under natural conditions marked by nitrogen and phosphorus limitations for biofuel and bioproduct production [61]. Similarly, *Tetraselmis suecica* (Chlorophyta) exhibited lower performance, achieving biomass productivity of 100 to 200 mg·L^−1^·d^−1^ and lipid productivity ranging from 5 to 8 mg·L^−1^·d^−1^ [62]. *Auxenochlorella pyrenoidosa* demonstrated a lipid productivity of 13.48 mg·L^−1^·d^−1^ when cultivated under acetate and ammonium conditions [63]. These comparisons showed that *M. gracile* displayed a superior capacity for growth and lipid accumulation under inorganic stress conditions, positioning it as a highly promising candidate for biofuel production compared to other microalgal species commonly utilized in the industry. A previous report suggested *Auxenochlorella* (formerly *Chlamydomonas*) as an alternative biotechnological tool for the bioremediation of wastewater [64]. However, one drawback was that the rate of hydrogen production was affected by the size of the microalgal cells. Further investigation into specific microalgal strains is essential for optimizing photobioreactor design, cultivation conditions, and wastewater treatments to enhance bioproduct production. Furthermore, the increased influx of CO_2_ into the photobioreactor complicated the regulation of its release from the cultivation medium and impacted the effectiveness of microalgae for large-scale cultivation. Therefore, our study explored the use of inorganic carbon as an alternative option that remains unaffected in terms of open-system large-scale biomass production.

Microalgae can accumulate lipids, which are used in the food, energy, cosmetics, and pharmaceutical industries [65]. Microalgae are characterized by their robust, thick cell walls, which limit the release of bioactive compounds due to the structural rigidity of the microalgal matrix [40]. Employing more environmentally friendly and effective techniques for lipid extraction from microalgae will enhance and broaden their potential uses [66]. Lipid extraction is an important microalgal process [67]. The method of Bligh and Dyer is commonly used for lipid extraction and showed the highest yield in our experiments. The extraction of lipids is more straightforward from a thoroughly dried microalgal sample compared to a sample that has undergone only dewatering [68]. Various conditions studied for lipid extraction include ultrasonic-assisted methods for maximum lipid yield from *M. gracile* IFRPD 1061. Ultrasonic-assisted lipid extraction from *M. gracile* IFRPD 1061 achieved high yields, with improved algal solubility for effective extraction [69] using emergent extraction methodologies [40]. Lipid ultrasonic-assisted extraction from microalgae has been previously reported. Total lipid extraction yield from *Scenedesmus* sp. with hexane using an ultrasonic-assisted method increased from 0.76 to 1.31 g·L^−1^ [70]. Lipid extraction from *Nannochloropsis* sp. biomass using hexane and an ultrasonic-assisted method gave lipid recovery yield higher than high-pressure homogeneity [71]. Other methods for extracting lipids from microalgae were also reported. Previous results recorded lipid extraction from *A. pyrenoidosa* microalga with hexane after microwave-assisted transesterification [72], while a mixture of hexane and methanol gave the highest lipid extraction from *Tetraselmis* sp. [27].

However, lipid extraction from *Messastrum* sp. microalgae has not yet been reported. Our results of lipid extraction from *M. gracile* IFRPD 1061 dried-biomass showed that a mixture of hexane and methanol was an alternative and promising solvent for large-scale lipid and bioproducts. Hexane is widely recognized as the solvent of choice for large-scale lipid extractions due to its technical and economic benefits, particularly its high selectivity for lipids and cost-effectiveness. Ongoing exploration using mixtures of hexane and polar solvents continues to improve the efficiency of lipid extraction methods from microalgae on a larger scale [27]. The lipid extracts exhibited a green color to the naked eye due to the chlorophyll pigment from *M. gracile* mixed with the extracted lipids. During extraction with hexane, chlorophyll a was dissolved in the nonpolar solvent, while chlorophyll b was partitioned into the polar methanol [73]. These impurities may act as effective and functional supplements, but the drawbacks of lipid recovery for exclusive lipid use should also be considered. Wet biomass is one of the alternative options for direct extraction. Previous reports detailed lipid extraction from wet yeast *Yarrowia lipolytica* SKY-7 using N-lauroyl sarcosine as a biodegradable anionic detergent and petroleum diesel as the co-solvent that had no effect on fatty acid [74,75]. The method used for preparing dried biomass can greatly influence its biochemical integrity. Freeze-drying effectively preserves these substances, while oven-drying, although more cost-effective, may result in denaturation of the cellular structure, thereby compromising quality. Therefore, selecting the appropriate drying method is crucial depending on the intended application of the biomass. [39,76]. Lipid accumulation in microalgae was also affected by extraction yields lower than 80%, which depended on total lipid recovery.

Traditional solvent-based techniques, such as methanol/hexane extraction in our study and co-extraction, continue to be highly effective for lipid extraction from microalgae. These methods can achieve lipid recovery rates of up to 90–95% and efficiently extract polar and nonpolar lipids. They are economically advantageous, requiring lower initial investments and simplifying scalability. Well-established protocols and infrastructure for solvent recovery and recycling support this, enhancing their economic feasibility for large-scale biofuel production [77]. However, these techniques also present environmental challenges due to the use of toxic solvents, with the need for solvent recovery and disposal raising significant environmental concerns [78]. By contrast, emerging green extraction techniques, such as supercritical CO_2_ extraction and ultrasound-assisted extraction, provide a more environmentally friendly alternative by minimizing or eliminating the use of hazardous solvents. These methods generally yield lower extraction efficiency and consume more energy for specific lipids, which hinders their competitiveness in large-scale applications [79]. They are also more expensive, complex, and less established for industrial biofuel production [80]. Green extraction methods show promise and may eventually become more cost-effective and efficient while, currently, traditional solvent-based techniques remain a widely used and practical solution for biofuel production from microalgae.

## 5. Conclusions

Our results showed that *Messastrum gracile* IFRPD 1061 effectively accumulated biomass and lipids using sodium carbonate and bicarbonate, achieving optimal yields of 392 mg·L^−1^·d^−1^ and 53.6 mg·L^−1^·d^−1^, respectively. Lipid extraction with methanol/hexane from freeze-dried biomass matched the control results, while methanol/hexane/chloroform worked well for oven-dried samples. However, challenges remain including the scalability of production, the environmental impacts of organic solvents, and the sustainability of inorganic carbon sources. Large-scale microalgae cultivation requires careful consideration of system design including reactor type, nutrient supply strategies, light penetration, and carbon source delivery. Efficient harvesting and dewatering techniques must be optimized to reduce costs and energy consumption. The integration of renewable energy sources and the development of closed-loop nutrient recycling systems will also be crucial for enhancing sustainability. Future research should explore alternative carbon sources and more eco-friendly solvents, along with conducting a life-cycle assessment to evaluate the overall feasibility of commercializing *M. gracile*. Addressing these design considerations will be essential for transitioning from laboratory-scale research to economically viable large-scale production.

## Figures and Tables

**Table 1:** Kinetic parameters of *Messastrum gracile* IFRPD 1061 microalgae cultivation under different conditions.

Condition (g·L^−1^)	$\mu_{m}$ (d^−1^)	$Q_{X}$ (mg·L^−1^·d^−1^)	$Q_{P}$ (mg·L^−1^·d^−1^)
Sodium carbonate induced			
1	0.54 ± 0.023 ^a^	195.03 ± 0.006 ^d^	30.10 ± 0.008 ^b^
2	0.53 ± 0.006 ^a^	336.34 ± 0.013 ^b^	33.86 ± 0.002 ^b^
3	0.52 ± 0.008 ^a^	256.55 ± 0.001 ^c^	40.00 ± 0.007 ^ab^
Sodium bicarbonate induced			
1	0.51 ± 0.013 ^a^	201.00 ± 0.001 ^d^	53.57 ± 0.007 ^a^
2	0.52 ± 0.026 ^a^	192.23 ± 0.009 ^d^	29.58 ± 0.003 ^b^
3	0.54 ± 0.001 ^a^	392.64 ± 0.015 ^a^	37.12 ± 0.007 ^b^

Data were calculated from triplicate experimental values ± standard deviation (SD). Different superscripts in the same column indicate a significant difference (*p* < 0.05).

**Table 2:** Lipid extraction yields from *Messastrum gracile* IFRPD 1061 under different conditions.

Condition	Extraction Yield (%)
Oven-dried biomass preparation	
(1) methanol/chloroform/water	44.31 ± 0.182 ^bc^
(2) methanol/hexane	42.18 ± 0.153 ^cd^
(3) hexane	21.62 ± 0.048 ^e^
(4) methanol/hexane/water	43.62 ± 0.016 ^bc^
Freeze-dried biomass preparation	
(1) methanol/chloroform/water	49.75 ± 0.234 ^a^
(2) methanol/hexane	46.81 ± 0.141 ^ab^
(3) hexane	20.22 ± 0.085 ^e^
(4) methanol/hexane/water	39.71 ± 0.038 ^d^

Data were calculated from triplicate experimental values ± standard deviation (SD). Different superscripts in the same column indicate a significant difference (*p* < 0.05).

## Data Availability

All original contributions are included in this article. Further inquiries can be directed to the corresponding author.

## Abbreviations

The following abbreviations are used in this manuscript:

$\mu_{m}$	Maximum specific growth rate (d^−1^)
$Q_{X}$	Volumetric productivity of biomass production (mg·L^−1^·d^−1^)
$Q_{P}$	Volumetric productivity of lipid production (mg·L^−1^·d^−1^)
